# Dynamic assessing silica particle-induced pulmonary fibrosis and associated regulation of long non-coding RNA expression in Wistar rats

**DOI:** 10.1186/s41021-021-00193-3

**Published:** 2021-06-15

**Authors:** Linlin Sai, Xuejie Qi, Gongchang Yu, Juan Zhang, Yuxin Zheng, Qiang Jia, Cheng Peng

**Affiliations:** 1 School of Public Health, Qingdao University, 308 Ningxia Road, Qingdao, 266071 Shandong China; 2 Department of Toxicology, Shandong Academy of Occupational Health and Occupational Medicine, Shandong First Medical University & Shandong Academy of Medical Sciences, 18877 Jingshi Road, Lixia District, Ji’nan, 250062 Shandong China; 3 Queensland Alliance for Environmental Health Science (QAEHS), The University of Queensland, Queensland, Australia

**Keywords:** Silicosis, Fibrosis, LncRNA, STEM, Rat

## Abstract

**Background:**

Exposure to respirable crystalline silica (RCS) can induce accelerated silicosis (AS), a form of silicosis that is more progressive and severe form of silicosis. In this project we aimed to assess processes of silicosis in rats exposed to RCS with focus on the regulation of long noncoding RNAs (lncRNAs).

**Results:**

The results showed that RCS induced acute inflammatory response as indicated by the appearance of inflammatory cells in the lung from the first day and peaked on day 7 of exposure. The fibroblasts appeared along with the inflammatory cells decreasing gradually on day 14. Extensive fibrosis appeared in the lung tissue, and silicon nodules were getting larger on day 28. Interestingly, the number of altered lncRNAs increased with the exposure time with 193, 424, 455, 421 and 682 lncRNAs on day 1, 7, 14, 21, and 28 after exposure, respectively. We obtained 285 lncRNAs with five significant temporal expression patterns whose expressions might correlate with severity of silicosis. KEGG analysis showed that lncRNAs from short time-series expression miner (STEM)-derived data mainly involved in 17 pathways such as complement and coagulation cascades.

**Conclusions:**

The differential expression profiles of lncRNAs may be potential biomarkers in silicosis through modulating expressions of their relevant genes in lungs of rat and thus warrant further investigation.

**Supplementary Information:**

The online version contains supplementary material available at 10.1186/s41021-021-00193-3.

## Introduction

Silicosis is an irreversible lung disease with pulmonary diffuse fibrosis as main manifestation resulting from long-term inhalation of occupational dust containing silica particles [1]. During the recent outbreaks of silicosis across Australia since 2015, about 350 silicosis patients have been diagnosed, and many were characterized as accelerated silicosis (AS) which is more rapidly progressive than traditional chronic silicosis [2]. AS is caused by inhalation of high intensity of RCS from artificial or engineered stones which contain around 90% crystalline silica, much higher than natural stones such as marble and granite [3]. Compared with traditional silicosis, accelerated silicosis is characterized by severe alveolitis, collagen deposition, and a progressive clinical course that often results in death shortly thereafter [4]. Because of the exposure to RCS from artificial stone, new cases of AS have kept re-emerging in developed countries as well as developed countries such as Australia, Israel and USA [5]. AS is more aggressive and severe in disease development and shorter latency periods [6].

Silicosis is characterized by excessive alveolar epithelial cell injury, abnormal inflammatory response, the aggregation of fibroblasts, extracellular matrix (ECM) accumulation, and epithelial-mesenchymal transition (EMT) [7, 8]. So far, the molecular mechanism of silicosis, especially AS has not been clearly understood yet, which hindered the effective treatment and prevention. This highlights the urgent need to explore the early response to RCS at different levels in initiation and key factors in progression and development of silicosis to identify the possible reliable biomarkers for early diagnosis or monitoring disease status.

LncRNA is a kind of non-protein-coding transcript with a length of more than 200 nucleotides, which is mainly distributed in the nucleus and cytoplasm and performs its functions conservatively in different ways [9]. LncRNAs are important signal transduction regulators that act in various patterns. Currently, increasing studies showed that lncRNAs exhibit significant regulatory functions in imprinting control, cell differentiation, and immune responses [10–13]. LncRNAs are capable of regulating transcription silencing and activation of protein-coding genes, associating with proteins to modulate their functions, and mRNAs to impact their translation, as well as acting as competing endogenous RNA (ceRNA) to suppress miRNAs function [14, 15]. Nowadays, the development of lncRNA microarrays has facilitated the research of noncoding RNAs (ncRNAs) in human diseases [13, 16, 17].

Moreover, emerging evidences showed the involvement of lncRNAs in silicosis and differential expression of lncRNAs was related to the development of silicosis. For instance, lncRNAs uc.77 and 2700086A05Rik have been shown to regulate EMT in the mouse model of pulmonary fibrosis [18]. LOC103691771 gene silencing attenuated myofibroblast differentiation and played a major role in myofibroblast differentiation induced by TGF-β1, which may serve as a potential therapeutic target for silicosis [19]. LncRNA CHRF can remove the inhibition of miR-489 on target genes MyD88 and Smad3 by absorbing miR-489, thereby activating fibrosis related signaling pathways and promoting the occurrence and development of pulmonary fibrosis [20]. Non-coding gene co-expression analyses implied that the different lncRNAs might play a role in silicosis through modulating expressions of the related mRNAs [21, 22]. In summary, investigation of expressions of lncRNA provide more functional information in pathogenesis of silicosis.

In this study, we assessed the RCS-induced silicogenic process at five time points and explored in parallel the role of aberrant expressions of lncRNAs in silicogenesis. We used lncRNA microarrays measure the dynamic lncRNA expression profiles in lung tissue of rats exposed to silica and identified a set of lncRNAs that were differentially expressed at different time points. Short time-series expression miner (STEM) analysis was used to reveal the changed trend of a collection of lncRNAs whose expression might correlate with disease activity of silicosis patients at different periods.

## Materials and methods

### Animals and treatment

Male Wistar rats at 5–7 week of age, weighting 180–200 g were obtained from Beijing Vital River Laboratory Animal Technology Co., Ltd. The proper care and use of these animals were following the institutional and national guidelines. The rats were housed in a temperature-controlled room (22 ± 2 °C) and a relative humidity of 55 ± 5%, with 12 h light-dark cycles and free access to water and chow. Food (manufactured by Laboratory Animal Center of Shandong Province, Shandong, China) and tap water were provided ad libitum. The SiO_2_ dust particle were purchased from Sigma-Aldrich (St. Louis, MO, USA). Silicon dioxide accounts for 99.7% of its chemical composition. The 80% diameter was between 1 and 5 μm. Silica particles were heated at 120 °C for 2 h to inactivate potential contaminating endotoxins and suspended in sterile saline at the concentration of 50 mg/ml. After acclimated to the environment for 2 weeks, 30 rats were randomly divided into two groups, a control group and a silica-exposed group with 15 animals each. Compared with traditional silicosis, AS can be caused by exposure to higher level of silica dust in short time [23]. In animal models, AS is induced by intratracheal instillation of large doses of silica [24]. Subsequently, silica-exposed groups were treated with intratracheal installation of 50 mg/ml silica suspension (1 ml per rat). The rats in the control group received the same volume of saline instead. The rats were weight and sacrificed on day 1, 7, 14, 21 and 28 after instillation, the lungs were harvested and weighted, and then stored at − 80 °C immediately for further analysis. The lung coefficient was calculated as lung coefficient = (lung wet weight/body weight) × 10^2^. The right lung tissues were used to hematoxylin and eosin (HE) and Masson staining. The left was used to analyze lncRNAs expression by microarray chips and their different expression trend by STEM analysis in lung tissues of rats at different time points.

### Histopathologic examination

The rats from each group were sacrificed by exsanguination under CO_2_ anesthesia after 1, 7, 14, 21, and 28 days, and the lung tissues were collected and stored at − 80 °C immediately. The right lung tissues were inflated with a 10% neutral buffered formalin solution overnight and rehydrated with 70% ethanol, and then embedded in paraffin before sectioning into 5 μm-thick slices. The slide sections were stained with HE and Masson trichrome to assess the degree of fibrosis. After HE and Masson staining, the morphological structure of lung tissues of rats in each group were observed under light microscope. The scoring criteria of Ashcroft [25] was used to evaluate the degree of pulmonary fibrosis in each group and allotted a score between 0 and 8 (Table 1).

**Table 1:** Criteria for grading lung fibrosis

Grade of fibrosis	Histological features
0	Normal lung
1	Minimal fibrous thickening of alveolar or bronchiolar walls with a few of inflammatory cells
2	Between 1 and 3
3	Moderate thickening of walls without obvious damage to lung architecture
4	Between 3 and 5
5	Increased fibrosis with definite damage to lung structure and formation of fibrous bands or small fibrous masses
6	Between 5 and 7
7	Severe distortion of structure and large fibrous areas; Typical silica nodules formed by collagen fibers.
8	Total fibrous obliteration of the field

### RNA extraction and microarray

Total RNA was extracted from 3 rats of silica-exposed group at five time points and 3 rats of control group using the Agilent Rat lncRNA array 8 × 60 K according to the manufacturer’s instructions. The yield of RNA was determined using a NanoDrop 2000 spectrophotometer (Thermo Scientific, USA) and the integrity was evaluated using agarose gel electrophoresis stained with ethidium bromide. After RNA isolation from the samples, the labeling rection, microarray hybridization, and washing were performed according to the Arraystar lncRNA Array protocol. Then the total RNA was transcribed to double strand cDNA, synthesized into cRNA and labeled with Cyanine-3-CTP onto the microarray. Finally, the arrays were scanned by the Agilent Scanner G2505C. The raw data was used to analyze array images using Agilent Feature Extraction Software and conduct the basic analysis using Gene spring (version 14.8, Agilent Technologies), and normalized with the quantile algorithm. Both fold change (FC) and *P* value of 3 silica-exposed rats and the 3 control rats were calculated from the normalized intensity. The threshold set for up- and down-regulated lncRNAs was set by a FC ≥ 2 and *P* ≤ 0.05.

### Short time-series expression miner analysis

The total lncRNAs were extracted from the rats. STEM clustering which is specifically designed to handle short time-series gene expression profiles was used to determine the significant temporal patterns in S-phase-derived lncRNAs. This method assumes the values of gene expression relative to a base time point. Then the clustering algorithm assigns each gene passing the filtering criteria to the model profile. The significant temporal expression patterns were obtained from expression profiles of S-phase lncRNAs at five different time points (day 1, 7, 14, 21 and 28).

### Real-time quantitative PCR validation

We selected 7 lncRNAs based on FC, *P* value and STEM clustering trend to further validate the expression of lncRNAs in control and silica-exposed rats at five time points measured by microarray using Q-RT-PCR. Q-RT-PCR was performed with a two-step reaction process: reverse transcription (RT) and PCR. Each RT reaction has two steps. In the first step is the mixture containing 0.5 μg RNA, 2 μL of 4 × gDNA wiper Mix, with Nuclease-free H_2_O up to 8 μL. Reactions were performed in a GeneAmp® PCR System 9700 (Applied Biosystems, USA) for 2 min at 42 °C. The second step is reaction with 2 μL of 5 × HiScript II Q RT SuperMixIIa performed in a GeneAmp® PCR System 9700 (Applied Biosystems, USA) for 15 min at 50 °C; 5 s at 85 °C. The 10 μL RT reaction mix was then diluted × 10 in nuclease-free water and held at − 20 °C. Real-time PCR was performed using LightCycler® 480 II Real-time PCR Instrument (Roche, Swiss) with 10 μL PCR reaction mixture that included 1 μL of cDNA, 5 μL of 2 × ChamQ SYBR qPCR Master Mix, 0.2 μL of forward primer, 0.2 μL of reverse primer and 3.6 μL of nuclease-free water. Reactions were incubated in a 384-well optical plate (Roche, Swiss) at 95 °C for 30 s, followed by 40 cycles of 95 °C for 10 s, 60 °C for 30 s. Each sample was run in triplicate for analysis. At the end of the PCR cycles, melting curve analysis was performed to validate the specific generation of the expected PCR product. The primer sequences were designed in the laboratory and synthesized by Generay Biotech (Generay, PRC) based on the mRNA sequences obtained from the NCBI database (Table 2). The expression levels of mRNAs were normalized to ACTB and were calculated using the 2-ΔΔCt method [26].

**Table 2:** The primers used in the present study

Gene Symbol	Forward primer (5 → 3)	Reverse primer (5 → 3)
ENSRNOT00000033123	ACAAGCATGATTCCTCCG	AATGTTGCCGTTCTCGAT
NONRATT029249.2	CGGATGCAGATCCGTCTCTA	GGAAGAGGAAAGGAAGTCAAC
NONRATT027882.2	ATCACTTACCATGAAATGGACC	CGACTAACCACTTTGCAGAG
NONRATT027881.2	CAGAACTGTAATCCAGAGCCAA	CTACACCTGCTCACCCAT
NONRATT014552.2	TGCTTACACAGACTCCACT	GGACACAACTTCATAGCACC
NONRATT009189.2	TTTACGGTCAGGCAGTTGT	TGGTGACTGAGAGATTGTCC
NONRATT018613.2	GAGGACAGGGATGGATAGG	TGAAGGAACCATCTGGGC

### KEGG pathway and GO analysis

The Kyoto Encyclopedia of Genes and Genomes (KEGG) pathway bioinformatics resource was used to analyze the enrichment pathway of lncRNA-associated binding sites target genes [27–29]. In this study, KEGG was used to analyze the lncRNAs which were screened by STEM clustering. Gene Ontology (GO) analysis was applied to analyze the function of such genes including biological process, cellular component, and molecular function. The statistics calculation of *P* value and enrichment of KEGG pathway were similar to the GO analysis, and the cut-off *P* value was set at 0.05.

### Statistical analysis

Data statistical analyses were performed using SPSS 20.0 software. Statistical probability of *P* ≤ 0.05 was considered statistically significant. Shapiro-Wilk test was used to test data for normality and Bartlett’s test for homogeneity of variance. Student t-test was used to compare the significant difference of body weights between control and silica-exposed groups. All data are shown as mean ± SD.

## Results

### Physiological and histopathological changes in rats

**Fig. 1:**
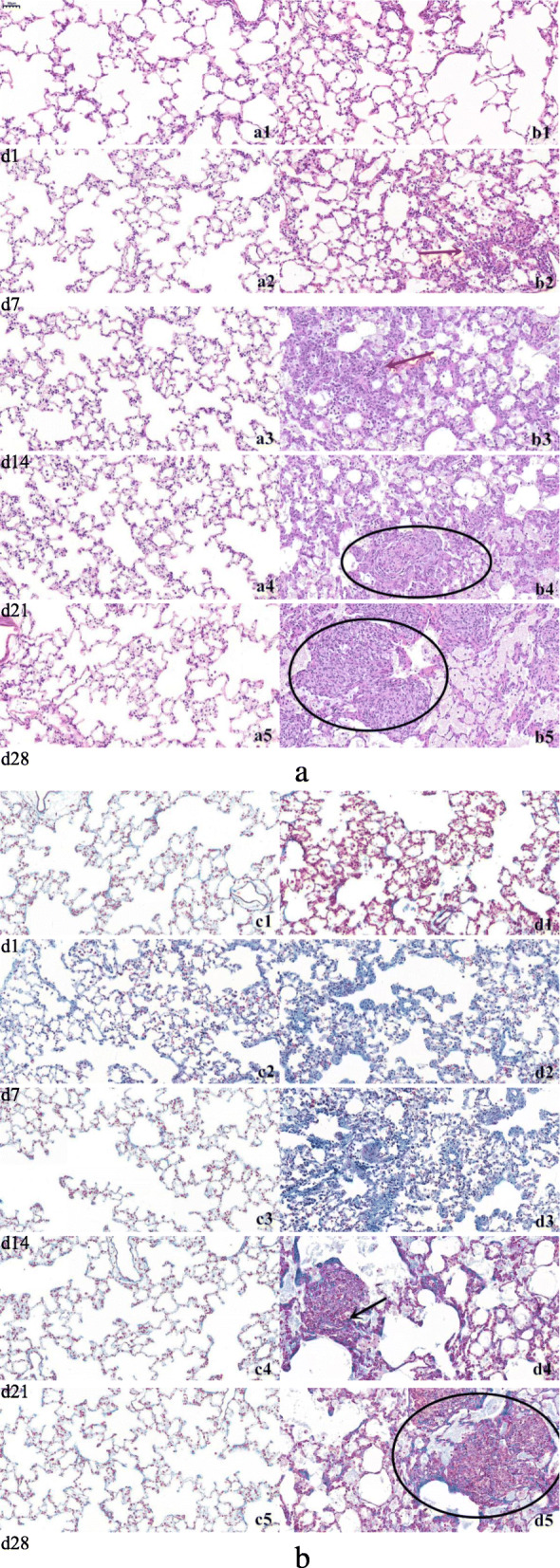
Histological examinations of lung of rats from control and silica-exposed groups at different time points. **a** HE staining for histopathologic changes in lungs of rats (× 200). a1-a5: Lungs from rats of control group on day 1, 7, 14, 21, 28, respectively; b1: Lungs of rats from silica-exposed group. A few inflammatory cells were found on day 1; b2-b3: Lungs of rats from silica-exposed group on day 7 and 14. Inflammatory cells (red arrow) increased; b4-b5: Lungs of rats from silica-exposed group on day 21 and 28. A large amount of consolidation areas and nodes (circle) formed; **b** Masson staining for histopathologic changes in lungs of rats (× 200). c1-c5: Lungs of rats from control group at day 1, 7, 14, 21, 28, respectively); d1-d2: Lungs of rats from silica-exposed group on day 1 and 7. There were normal lung tissue with no visible fibrous tissue; d3: Lungs of rats from silica-exposed group on day 14. A few of fibroblast were observed; d4: Lungs of rats from silica-exposed group on day 21. Fibroblast gradually increase and tiny collagen fibers (black arrow) were found; d5: Lungs of rats from silica-exposed group on day 28. Dense deposits of blue collagenous fibers and primary cellular nodules (circle) were observed in the interstitium of the lungs from rats

In this study, we examined rats with and without exposure to silica dust on day 1, 7, 14, 21, 28 after exposure to high-dose of SiO_2_ and then observed their body weight and lung coefficient. We observed significant changes in body weight of rats in the silica-exposed group on day 28 (*P* ≤ 0.05) and the lung coefficient silica-exposed rats were significantly increased when compared to controls on day 28 (*P* < 0.01) [22]. In animal models, both accelerated silicosis with high-dose silica and chronic silicosis with low-dose silica exposures induce granulomatous changes in the lung [30, 31]. The granuloma-like structures in accelerated silicosis are loosely aggregated foamy histiocytes [32]. In present study, we examined the dynamic change from inflammation to fibrosis in acute silicosis. In the control rats, HE and Masson staining under microscope showed a clear structure of the right lung tissues, integrated alveolar walls and no obvious inflammatory infiltration at each time point. However, in the lung of rats from silica-exposed group, HE staining showed that there were few inflammatory cells in the lung tissue, and the alveolar septum was slightly widened after 1 day exposure. Masson staining showed normal lung tissue with no visible fibrous tissue on day 1. The pathology grade was scored as 1. On day 7, there were inflammatory cells infiltrating mainly with neutrophils in the alveoli of rats with exudates. The pathology grade is defined as 3. On day 14, the alveolar septa is widened and part of the septa is destroyed, and the alveolar cavity is narrowed. Meanwhile fibroblast were observed with the pathology grade defined as 5. On day 21, the exudate dominated by inflammatory cells decreased. But fibroblasts significantly increased. The damaged alveolar structure of the lung tissue was observed with less number of necrotic cells. Masson stained showed that collagen fibers begins to increase. The pathology grade is defined as 7. On day 28, the alveolar structure is largely destroyed and typical silica nodules were formed. Dense deposits of blue collagenous fibers and primary cellular nodules were observed in the interstitium of the lungs from rats, and collagen hyperplasia was evident around local granulation tissue, bronchi and vascular walls. The pathology grade is defined as 8 (Fig. 1).

### Overview of lncRNA and mRNA profiles

The expression of lncRNAs measured by the microarray in lung tissues of rats showed significant difference between silica-exposed groups and controls at five time points (FC ≥ 2, *P* ≤ 0.05). On day 1, up to 193 differentially expressed lncRNAs were detected, in which 73 lncRNAs were up-regulated and 120 lncRNAs were down-regulated. On day 7, 424 differentially expressed lncRNAs were detected, in which 244 lncRNAs were up-regulated and 180 lncRNAs were down-regulated. On day 14, 455 differentially expressed lncRNAs were detected with 192 up-regulated lncRNAs and 263 down-regulated lncRNAs. On day 21, 421 differentially expressed lncRNAs were detected, including 203 up-regulated lncRNAs and 218 down-regulated lncRNAs. On day 28, 682 differentially expressed lncRNAs were detected, in which 300 lncRNAs were up-regulated and 382 lncRNAs were down-regulated (Fig. S1). The top 10 up-regulated and 10 down-regulated lncRNAs at five time points with the highest fold change were summarized (Table S1–5).

**Fig. 2:**
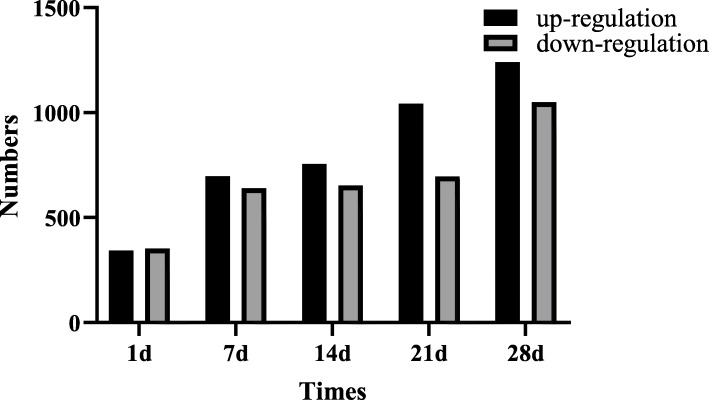
Bar graph of the number of differentially expressed mRNAs at five times points. The abscissa represents the different time points (day 1, 7, 14, 21, 28 after treatment). The ordinate represents the number of differentially expressed mRNAs

Meanwhile, the differential expression of mRNAs were also measured in lung tissues between silica-exposed groups and controls at five time points (FC ≥ 2, *P* ≤ 0.05). On day 1, up to 696 differentially expressed mRNAs were detected, in which 343 mRNAs were up-regulated and 353 mRNAs were down-regulated. On day 7, 1336 differentially expressed mRNAs were detected, in which 697 mRNAs were up-regulated and 639 mRNAs were down-regulated. On day 14, 1409 differentially expressed mRNAs were detected with 756 up-regulated mRNAs and 653 down-regulated mRNAs. On day 21, 1737 differentially expressed mRNAs were detected, including 1042 up-regulated mRNAs and 695 down-regulated mRNAs. On day 28, 2289 differentially expressed mRNAs were detected, in which 1240 mRNAs were up-regulated and 1049 mRNAs were down-regulated (Fig. 2).

### Silicosis activity-dependent lncRNAs clustering

**Fig. 3:**
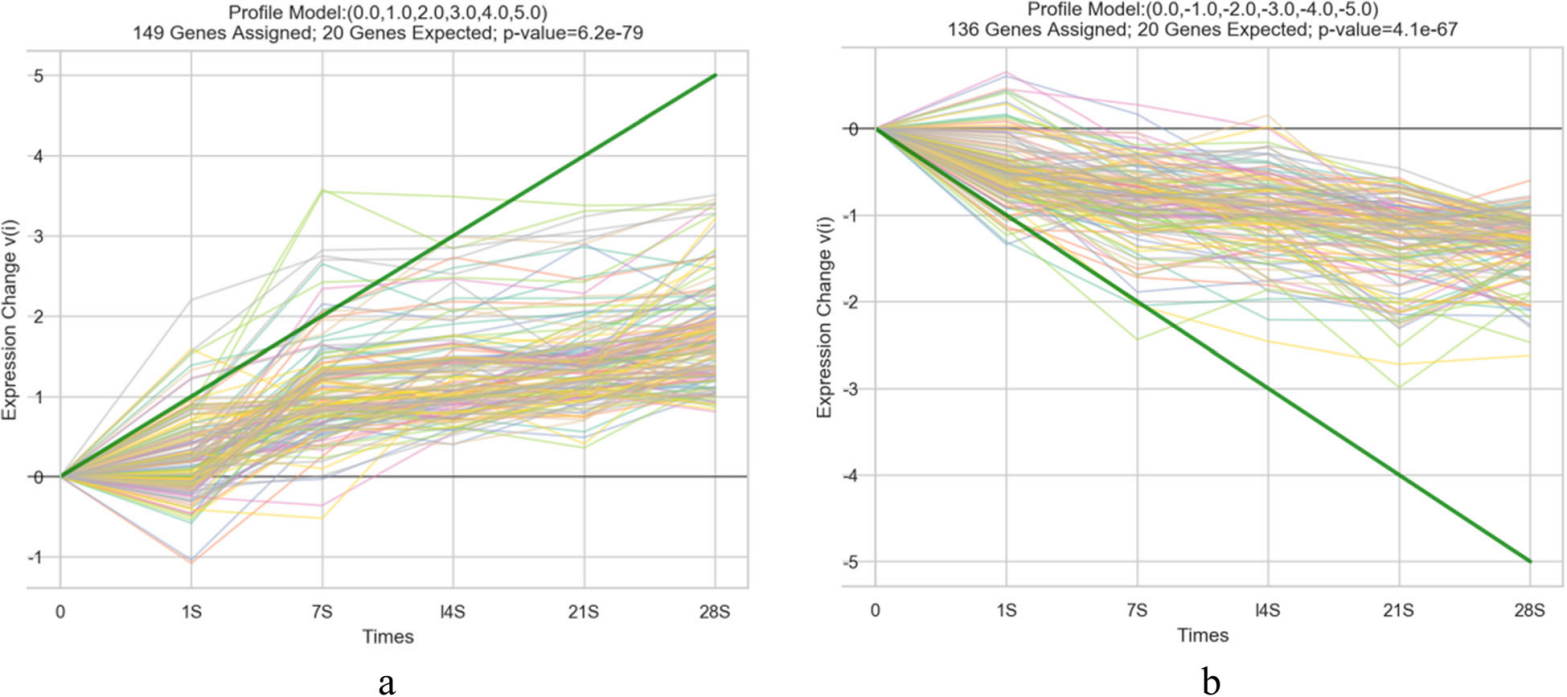
LncRNAs clustering by short time series expression miner analysis. **a** The result of profile 39. **b** The result of profile 8. Each line in the figure represents an expression value of the corresponding lncRNAs. The abscissa represents the different time points (day 1, 7, 14, 21, 28 after treatment). The ordinate represents the 10log2 value of the fold change. Negative values indicate down-regulated expression, and positive values indicate up-regulated expression

According to STEM clustering, all the lncRNAs were classified into 50 clusters (profile 0 - profile 49) based on their different expression trend at five time points (Fig. S2). The colored profiles have a statistically significant number of genes assigned and the same colored profiles are all similar to each other. The result showed that 9 clusters were statistically significant (*P* < 0.05) among which two profiles (profile 39, profile 8) were selected according to their total trend (Fig. 3). The profile 39 which has with the smaller (*P* = 6.2 × 10^− 79^) and contained 149 lncRNAs, elevated at different time points. The profile 8 containing a total of 136 lncRNAs showed an overall decreasing trend.

### Validation of lncRNA expression by Q-RT-PCR

**Fig. 4:**
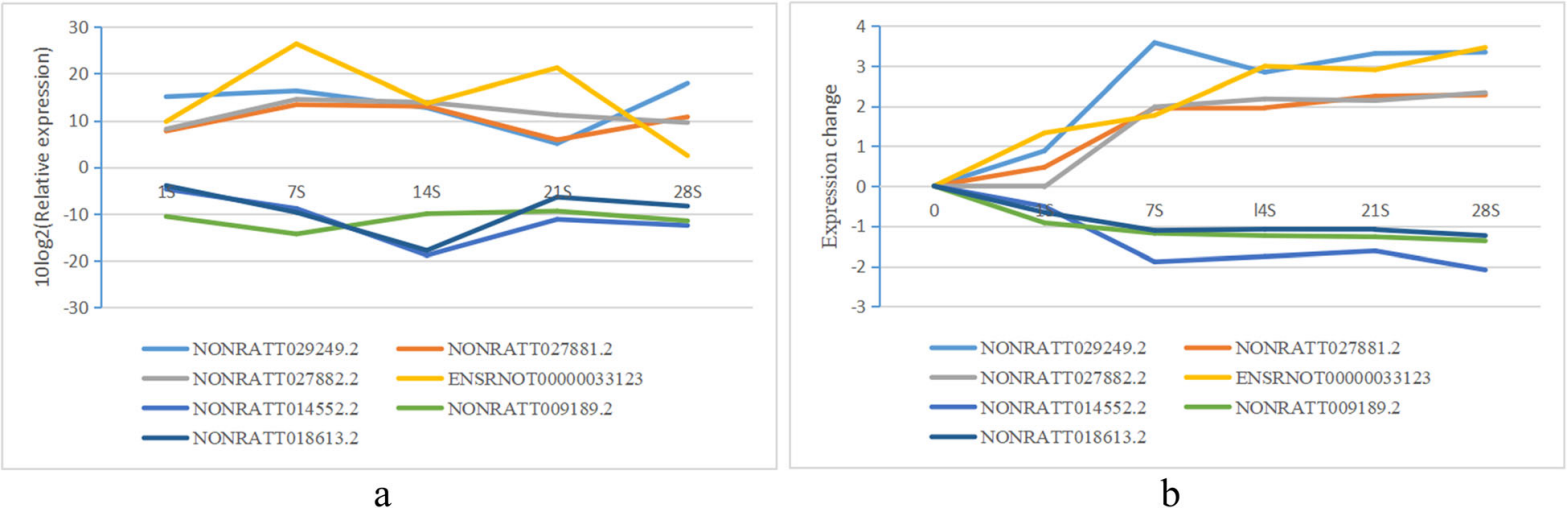
The differential expression of lncRNAs according to Q-RT-PCR and STEM analysis. **a** Line chart of the expression of lncRNAs by Q-RT-PCR. Each line in the figure represents a lncRNA. The abscissa represents the different time points (day 1, 7, 14, 21, 28 after treatment). The ordinate represents the 10log2 value of the relative expression. Negative values indicate down-regulated expression, and positive values indicate up-regulated expression. **b** Line chart of the expression of lncRNAs according to STEM analysis. Each line in the figure represents a lncRNA. The abscissa represents the different time points (day 1, 7, 14, 21, 28 after treatment). The ordinate represents the expression change value. Negative values indicate down-regulated expression, and positive values indicate up-regulated expression

Analysis of 7 lncRNAs by Q-RT-PCR showed that in lung tissue of rats exposed to silica 4 lncRNAs were up-regulated (NONRATT029249.2, NONRATT027881.2, NONRATT027882.2, ENSRNOT00000033123; FC ≥ 2; *P* ≤ 0.05) with rising expression trends in STEM analysis except NONRATT027882.2. Three down-regulated lncRNAs in silicosis of rats (NONRATT014552.2; NONRATT009189.2; NONRATT018613.2; FC ≥ 2; *P* ≤ 0.05), which were all decreased in STEM analysis (Fig. 4). This validation confirmed the good reproducibility and reliability of the data by microarray and STEM analysis.

### The enrichment pathways and terms of differentially expressed lncRNAs-associated target genes by KEGG and GO analysis

**Fig. 5:**
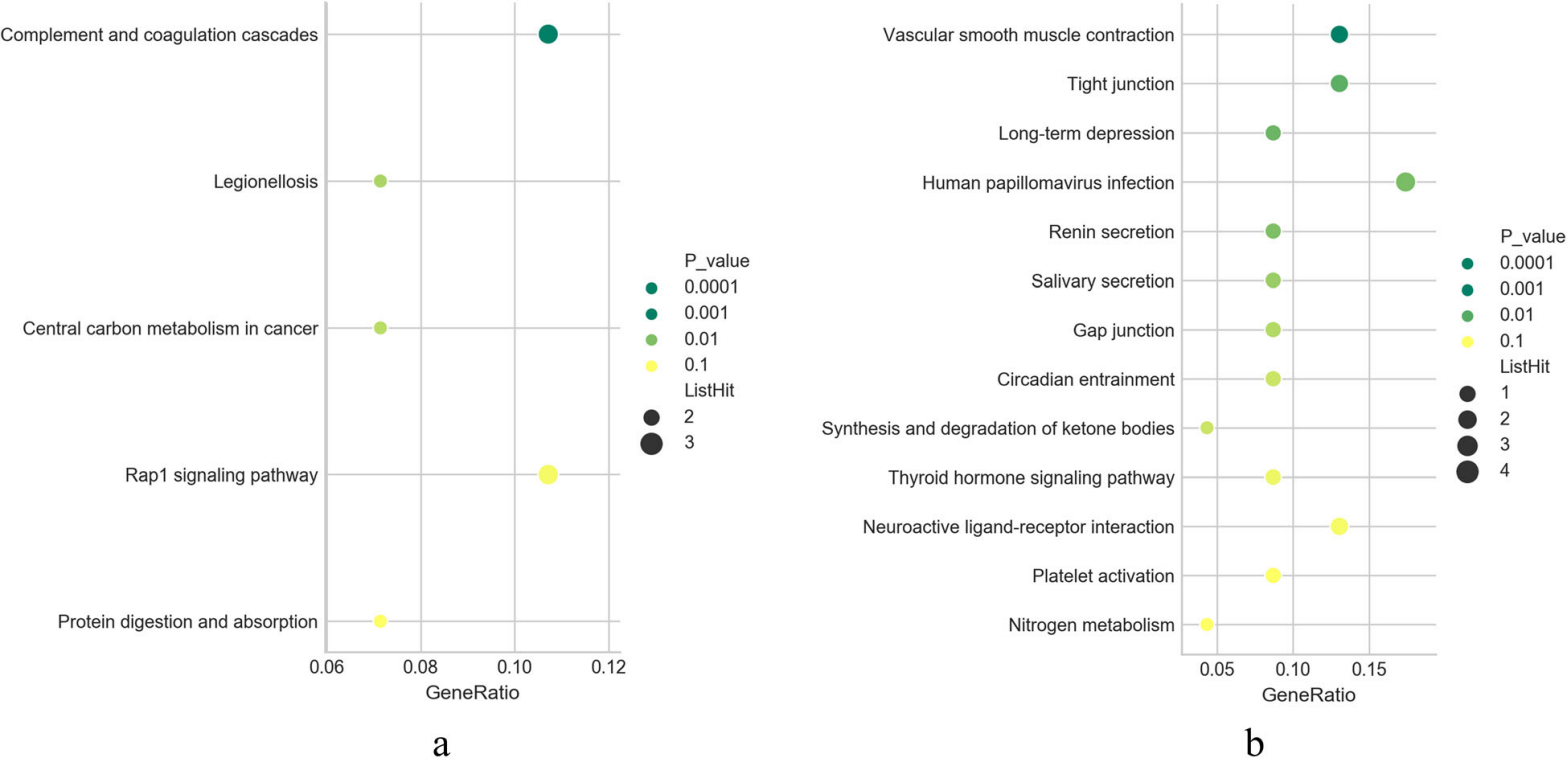
The bubble diagrams of KEGG analysis results. Abscissa represents the enrichment degree and ordinate represents the enrichment pathway. The larger the midpoint, the more genes that fall into the pathway, and the greener the color, the higher the enrichment significance. **a** The annotated significant pathways targeted by the differentially expressed lncRNAs from profile 39 of STEM. **b** The annotated significant pathways targeted by the differentially expressed lncRNAs from profile 8 of STEM

**Fig. 6:**
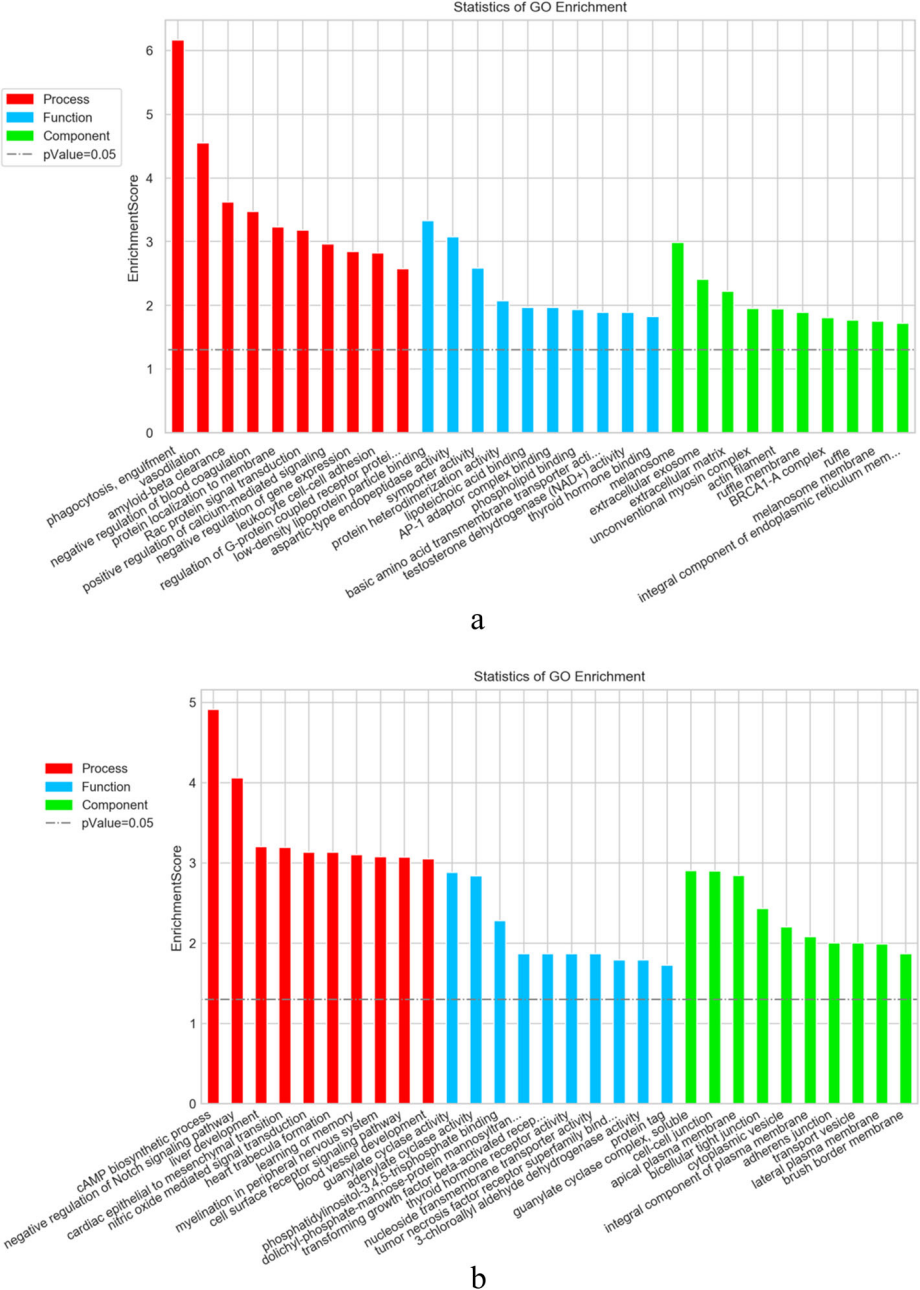
GO bar graph of differentially expressed lncRNAs. Ordinateis -log10 (*P* value). The higher the bar graph is, the smaller the corresponding *P* value is. Different color distribution corresponds to Biological Process, Cellular Component, Molecular Function, respectively. **a** GO categories from profile 39 of STEM. **b** GO categories from profile 8 of STEM

To identify the potential key pathways involved in pulmonary fibrosis, we analyzed the mRNA in the same chain of differentially expressed lncRNAs from profile 39 and profile 8 within the range of 50 kb using KEGG method. Data analysis indicated that 69 pathways were found in profile 39. We then acquired 5 pathways (*P ≤* 0.05) based on the *P* value (Fig. 5a). Seventy pathways were found in profile 8 and 13 pathways from acquired (*P ≤* 0.05) (Fig. 5b). GO enrichment analysis indicated that the different genes in profile 39 of STEM were mostly enriched 40 functional terms in its Molecular Function; 25 terms in Cellular Component; 125 terms in Biological Process. The different genes in profile 8 of STEM were mostly enriched 35 functional terms in its Molecular Function; 23 terms in its Cellular Component;158 terms in its Biological Process. The top 10 terms of each program of profile 39 and profile 8 were shown in Fig. 6.

## Discussion

Silicosis is one of the most serious occupational diseases causing high morbidity and mortality worldwide [33, 34]. AS is one of the clinical and pathologic varieties of silicosis that is caused by the inhalation of RCS [23, 35]. Some of cases of AS have progressed rapidly, suggesting that AS is more aggressive than classic silicosis [36]. However, the etiology of susceptibility and molecular mechanisms to AS is not well understood. The clinical irreversibility of pathogenic process and elusive pathogenic mechanisms underlying silicosis presents a great challenge to its effective treatment. Recent studies have revealed the novel functions of thousands of lncRNAs in inflammatory and lung diseases [15, 37], which prompted us to propose the hypothesis that lncRNAs may play a role in initiation and progress of RCS-induced accelerated silicosis.

To test the hypothesis, we developed silica-exposed rat model for 28 days to observe the histological changes in lung tissue of rats and examine the expression of lncRNAs in parallel at different time intervals during the latency period up to 28 days after exposure. These results of body weight and the lung coefficient of rats indicated that silica dust induced lung edema on day 28. Meanwhile, we speculated that significant lung injury may restrain body growth through the abnormal metabolism of silica-exposed rats.

The results of histopathological evaluation showed a clear time-dependent changes with inflammation and fibrosis in lung tissues of the rats exposed to silica dust. Lung inflammation and apoptosis are hallmarks of accelerated silicosis. However, diffusibility granulomas arise by chronic inhalation of silica without significant lung inflammation or apoptosis [31]. In our study, the inflammatory cells appeared from the first day and increased until day 14. Numerous inflammatory cells were recruited to the alveoli and led to incrassation of the alveoli walls. These results showed that silica particles increased inflammation and destroyed alveoli integrity. Then fibroblasts appeared along with the inflammatory cells decreasing gradually from day 14. This result showed that with Inflammation gradually subside over time, lung tissue begins to repair when the damage reaches a certain degree. Myofibroblasts are important effector cells in tissue repair. A few of collagen fibers begins to appear after 21 days and then gradually accumulated. Finally, the typical silicon nodules are formed gradually and the pulmonary fibrosis was obvious on day 28. This suggest that the formation of pulmonary fibrosis is an accumulation process and inhibiting the progression of pulmonary fibrosis is the fundamental measure to prevent the formation of silicosis. In brief, the pathological grade keep rising from day 1 to day 28. Zhao et al. examined pathological lung changes on day 1, 7, 28, 56 in mice which pathological damage was mild than our results [38]. There is an existing evidence that the lungs of mice that were subjected to intratracheal instillation of quartz had milder inflammatory changes than the lungs of quartz-treated rats [39]. Our study examined lung pathology on day 14, 21 in addition. And we predict that 14 days after silica-exposed is a critical time point for silicosis progression.

Furthermore, the expression profiles of lncRNAs in silica-induced rats and controls were detected at five time points. The results showed that the amount of differentially expressed lncRNAs were increased from day 1 to day 14. We predict that the amount of lncRNAs increased may associate to the aggravation of pulmonary inflammatory response. However, the number of lncRNAs decreased on day 21 when inflammation gradually subside and fibroblast accumulation. So we speculated that the changes of lncRNAs may relevant to the inflammation diminished and fibrosis enhanced. The differentially expressed lncRNAs increased with collagen fiber aggregation on day 28. In short, we supposed that the changes of the profiles of lncRNAs may closely relate to the development of silicosis and differentially expressed lncRNAs may be potential biomarkers of silicosis.

STEM analysis is a commonly used bioinformatics method to determine statistically significant time-dependent gene expression profiles [40, 41]. STEM implements unique methods to cluster, compare, and visualize short time series expression data according to different time points. This method assumes the values of a sort of expression represent log ratios relative to the expression at the first time point. Up to now, no data has been reported about the STEM analysis of lncRNA on day 1, 7, 14, 21, 28 in silica-exposed rats. Therefore, based on the results of the profile of differentially expressed lncRNAs, we proceeded the STEM analysis and the results showed that the level of 149 lncRNAs were increased in profile 39 and 136 lncRNAs were decreased in profile 8 over time. So we predicted that 149 lncRNAs in profile 39 may have close positive correlation with the development of silicosis, but 136 lncRNAs in profile 8 may have close negative correlation with the development of silicosis. Meanwhile, we found 28 and 23 mRNAs related to lncRNAs in profile 39 and 8 respectively. Based on the result of differently expressed mRNAs, we found that 26 mRNAs related to lncRNAs in profile 39 were up-regulated and 15 mRNAs related to lncRNAs in profile 8 were down-regulated (Table S6). The results implied that lncRNAs as ceRNA play an important role in the development of silicosis. As can be seen from the overall trend, these two groups of lncRNAs may have potential biomarkers of the development of silicosis.

Then we choose 7 differentially expressed lncRNAs (4 lncRNAs up-regulated and 3 lncRNAs down-regulated) with high value of FC and low value of *P* basing on the results of STEM analysis for Q-RT-PCR. Among them, the tendency of 6 lncRNAs were consisted with the result of STEM analysis. These data demonstrated that these 6 lncRNAs may possess important functions during pulmonary fibrosis induced by silicosis. Meanwhile, this validation confirmed the good reproducibility and reliability of the data by microarray. So far, no data has been reported about the pathway analysis of target genes related to lncRNAs in profile 39 and 8. Therefore, in the present study, we used KEGG pathway annotation method to analyze these lncRNA-associated target genes in the lungs of silica-exposed rats. Based on the result of STEM, we selected lncRNA-associated target genes in profile 39 and profile 8 to look for several key pathways that represent potential target pathways to the development of pulmonic damage. The result showed that 17 pathways may be associated with the occurrence and development of silica-induced pulmonary fibrosis in rat referencing MalaCard database and *P* value (Table 3). Ranking the *P* value, the top one is “Complement and coagulation cascades” signaling pathway. The complement system is a proteolytic cascade in blood plasma and a mediator of innate immunity, a nonspecific defense mechanism against pathogens [42]. ECM proteins as regulators of the complement system [43]. ECM has an important role in influencing immune cell behaviour in inflamed tissues. Meanwhile, it is related to the development of silicosis [44, 45]. In addition, coagulation of insoluble fibrin and the infiltration of inflammatory cells are the key and essential factors for fibrotic process [46]. Hence, the role of “Complement and coagulation cascades” signaling pathway in the development of silica-induced pulmonary fibrosis needs to further study.

**Table 3:** The pathways may be associated with pulmonary fibrosis in profile 39 and 8

Pathway	Profile	FoldEnrichment	*P*-value
Complement and coagulation cascades	39	10.81239243	0.002570713
Legionellosis	39	10.31527094	0.015871597
Central carbon metabolism in cancer	39	9.496598639	0.018560791
Rap1 signaling pathway	39	4.135615537	0.034897583
Protein digestion and absorption	39	6.364741641	0.038990204
Synthesis and degradation of ketone bodies	8	33.10671937	0.029811592
Nitrogen metabolism	8	20.23188406	0.048337941
Long-term depression	8	11.74754558	0.012341775
Renin secretion	8	10.71099744	0.014720198
Salivary secretion	8	9.337792642	0.019085892
Vascular smooth muscle contraction	8	8.810659187	0.004517326
Gap junction	8	8.276679842	0.023929619
Circadian entrainment	8	7.357048748	0.029777868
Tight junction	8	6.426598465	0.010793826
Thyroid hormone signaling pathway	8	6.172439204	0.041065765
Platelet activation	8	5.646107179	0.048232816
Human papillomavirus infection	8	4.161987578	0.014133566

In the result of KEGG pathway analysis, “Vascular smooth muscle contraction” signaling pathway was involved in silica-induced pulmonary fibrosis in rat. It has been accepted, in case of the vessel damage, vascular smooth muscle cells (VSMCs) are able to switch from the quiescent ‘contractile’ phenotype to the ‘pro-inflammatory’ phenotype. This change is accompanied by decrease in expression of production of pro-inflammatory mediators that modulate induction of proliferation and chemotaxis [47]. Apoptotic VSMCs release pro-inflammatory cytokines in surrounding non-apoptotic VSMCs and induce an inflammatory response [48]. Since inflammation is key to initiate lung fibrosis, it is not surprising that this signaling pathway is identified in our rat lung fibrosis model. Hence, we speculated that “Vascular smooth muscle contraction” signaling pathway may be play an important role in silica-induced pulmonary fibrosis of rats and worth further study.

“Tight junctions” signaling pathway was included in the result of KEGG pathway analysis. Ohta et al. found that TGF-β, a key regulator of fibrosis, disrupted tight junctions of epithelial and endothelial cells, indicating that TGF-β may partially disrupt tight junctions [49]. However, much less are known about the cross-talk between the tight junction signaling pathway and pulmonary fibrosis in rat.

Interestingly, we found the increased expression of “Central carbon metabolism in cancer” pathway which is involved in the metabolism of cancer cells especially cancer stem cells [50]. RARC has classified RCS as a human carcinogen [51]. Causal association between silicosis and lung cancer has been documented [52]. Alterations in cancer central carbon metabolism including aerobic glycolysis, elevated glutaminolysis, dysregulated TCA cycle and pentose phosphate pathway have been identified driver reactions in cancer by providing building biomass for proliferation [50]. Furthermore, metabolic processes have an essential role in the regulation of the cellular redox balance [53]. Therefore, our result provides molecular evidence of the important role of central carbon metabolism in driving silica particle-induced pulmonary fibrosis to cancer.

## Conclusions

Our results showed that rats exposed to silicon dioxide by disposable intratracheally instilled method were induced inflammation and pulmonary fibrosis from 1 to 28 days after exposure. Fibroblasts appeared along with the inflammatory cells decreasing gradually from day 14 which were considered as a crucial time point in silicosis progression. Finally, extensive fibrosis appeared and the typical silicon nodules formed gradually on day 28. The change of the number of differentially expressed lncRNAs may be related to the progression of pulmonary inflammation and fibrosis in rats. The result of STEM showed that 149 lncRNAs were increased and 136 lncRNAs decreased with five significant temporal expression patterns. The result of KEGG pathway analysis of these lncRNAs showed that “Complement and coagulation cascades”, “Vascular smooth muscle contraction”, “Tight junctions” and “Central carbon metabolism in cancer” signaling pathway may be associated with the development of silicosis. Importantly, the roles of these lncRNAs in STEM result which were involved in the four pathways need to be further investigated. More studies exploring the contribution of lncRNAs to silicosis are expected in future and will be critical for the deepening understanding and then effective therapy of this disorder.

## Supplementary Information

**Additional file 1: Fig. S1.** Heat map showing significant differentially expressed lncRNAs in lungs of rat model of pulmonary fibrosis. a: [1S3], [1S1] and [1S2] represent pulmonary fibrosis samples on the first day; [1C1], [1C2] and [1C3] represent control samples. b: Silica-exposed group and control samples on 7th day. c: Silica-exposed group and control samples on 14th day. d: Silica-exposed group and control samples on 21th day. e: Silica-exposed group and control samples on 28th day. Each row represents a lncRNA and each column represents a sample. Dendrograms produced by clustering analysis of the samples are shown on the top. The red represent up-regulated lncRNAs and blue represent down-regulated lncRNAs in lungs of rat model of pulmonary fibrosis compared to control. **Fig. S2.** The data from lung tissues of rats of silica-induced was sampled at five time points (day 1, 7, 14, 21 and 28). The colored profiles had a statistically significant number of genes assigned. Non-white profiles of the same color represent profiles grouped into a single cluster. **Table S1.** The top 10 up-regulated and down-regulated lncRNAs in lungs of silica-induced rats on the first day. **Table S2..** The top 10 up-regulated and down-regulated lncRNAs in lungs of silica-induced rats on the 7th day. **Table S3**. The top 10 up-regulated and down-regulated lncRNAs in lungs of silica-induced rats on the 14th day. **Table S4.** The top 10 up-regulated and down-regulated lncRNAs in lungs of silica-induced rats on the 21th day. **Table S5.** The top 10 up-regulated and down-regulated lncRNAs in lungs of silica-induced rats on the 28th day. **Table S6.** The up-regulated and down-regulated mRNAs related to lncRNAs in profile 39 and 8 in lungs of silica-induced rats.

## Data Availability

All generated data are included in this manuscript.

## Abbreviations

RCS: Respirable crystalline silica
AS: Accelerated silicosis
LncRNAs: Long noncoding RNAs
ECM: Extracellular matrix
EMT: Epithelial-mesenchymal transition
ceRNA: Competing endogenous RNA
ncRNAs: Noncoding RNAs
HE: Hematoxylin and eosin
STEM: Short time-series expression miner
Q-RT-PCR: Real-time quantitative PCR
RT: Reverse transcription
FC: Fold change
KEGG: Kyoto Encyclopedia of Genes and Genomes
GO: Gene Ontology
VSMCs: Vascular smooth muscle cells

## References

[1] Zhang Y, Wang F, Zhou D (2016). Genome-wide analysis of aberrantly expressed microRNAs in bronchoalveolar lavage fluid from patients with silicosis. Ind Health.

[2] Levin K, McLean C, Hoy R (2019). Artificial stone-associated silicosis: clinical-pathological-radiological correlates of disease. Respirol Case Rep.

[3] Fazen LE, Linde B, Redlich CA (2020). Occupational lung diseases in the 21st century: the changing landscape and future challenges. Curr Opin Pulm Med.

[4] Chong S, Lee KS, Chung MJ (2006). Pneumoconiosis: comparison of imaging and pathologic findings. Radiographics.

[5] Leso V, Fontana L, Romano R (2019). Artificial stone associated silicosis: a systematic review. Int J Environ Res Public Health.

[6] Hutyrová B, Smolková P, Nakládalová M (2015). Case of accelerated silicosis in a sandblaster. Ind Health.

[7] Rong Y, Shen Y, Zhang Z (2015). Blocking TGF-β expression inhibits silica particle-induced epithelial-mesenchymal transition in human lung epithelial cells. Environ Toxicol Pharmacol.

[8] Fang K, Liu P, Dong S (2016). Magnetofection based on superparamagnetic iron oxide nanoparticle-mediated low lncRNA HOTAIR expression decreases the proliferation and invasion of glioma stem cells. Int J Oncol.

[9] Sanchez Calle A, Kawamura Y, Yamamoto Y (2018). Emerging roles of long non-coding RNA in cancer. Cancer Sci.

[10] Spurlock CF, Tossberg JT, Guo Y (2015). Expression and functions of long noncoding RNAs during human T helper cell differentiation. Nat Commun.

[11] Zhang Y, Cao X (2016). Long noncoding RNAs in innate immunity. Cell Mol Immunol.

[12] Fitzgerald KA, Caffrey DR (2014). Long noncoding RNAs in innate and adaptive immunity. Curr Opin Immunol.

[13] Wang Y, Li Z, Zheng S (2015). Expression profile of long non-coding RNAs in pancreatic cancer and their clinical significance as biomarkers. Oncotarget.

[14] Shi Y, Wang Y, Luan W (2014). Long non-coding RNA H19 promotes glioma cell invasion by deriving miR-675. PLoS One.

[15] Yuan JH, Yang F, Wang F (2014). A long noncoding RNA activated by TGF-β promotes the invasion-metastasis cascade in hepatocellular carcinoma. Cancer Cell.

[16] Xu J, Gao C, Zhang F (2016). Differentially expressed lncRNAs and mRNAs identified by microarray analysis in GBS patients vs healthy controls. Sci Rep.

[17] Liu XY, Wang L, Yu B (2015). Expression signatures of long noncoding RNAs in adolescent idiopathic scoliosis. Biomed Res Int.

[18] Sun H, Chen J, Qian W (2016). Integrated long non-coding RNA analyses identify novel regulators of epithelial-mesenchymal transition in the mouse model of pulmonary fibrosis. J Cell Mol Med.

[19] Cai W, Xu H, Zhang B (2020). Differential expression of lncRNAs during silicosis and the role of LOC103691771 in myofibroblast differentiation induced by TGF-β1. Biomed Pharmacother.

[20] Wu Q, Han L, Yan W (2016). miR-489 inhibits silica-induced pulmonary fibrosis by targeting MyD88 and Smad3 and is negatively regulated by lncRNA CHRF. Sci Rep.

[21] Liang H, Pan Z, Zhao X (2018). LncRNA PFL contributes to cardiac fibrosis by acting as a competing endogenous RNA of let-7d. Theranostics.

[22] Sai L, Yu G, Bo C (2019). Profiling long non-coding RNA changes in silica-induced pulmonary fibrosis in rat. Toxicol Lett.

[23] Barnes H, Goh NSL, Leong TL (2019). Silica-associated lung disease: an old-world exposure in modern industries. Respirology.

[24] Borges VM, Lopes MF, Falcão H (2002). Apoptosis underlies immunopathogenic mechanisms in acute silicosis. Am J Respir Cell Mol Biol.

[25] Ashcroft T, Simpson JM, Timbrell V (1988). Simple method of estimating severity of pulmonary fibrosis on a numerical scale. J Clin Pathol.

[26] Livak KJ, Schmittgen TD (2001). Analysis of relative gene expression data using real-time quantitative PCR and the 2(−Delta Delta C(T)) method. Methods.

[27] Hu G, Wei B, Wang L (2015). Analysis of gene expression profiles associated with glioma progression. Mol Med Rep.

[28] Xing Z, Chu C, Chen L (2016). The use of gene ontology terms and KEGG pathways for analysis and prediction of oncogenes. Biochim Biophys Acta.

[29] Kanehisa M, Sato Y, Morishima K (2016). BlastKOALA and GhostKOALA: KEGG tools for functional characterization of genome and metagenome sequences. J Mol Biol.

[30] Castranova V, Porter D, Millecchia L (2002). Effect of inhaled crystalline silica in a rat model: time course of pulmonary reactions. Mol Cell Biochem.

[31] Nakano-Narusawa Y, Yokohira M, Yamakawa K (2020). Single Intratracheal quartz instillation induced chronic inflammation and tumourigenesis in rat lungs. Sci Rep.

[32] Sasi A, Ray A, Bhalla AS (2020). Chylothorax in a case of accelerated silicosis with pulmonary silicoproteinosis: a unique association. Indian J Occup Environ Med.

[33] Carneiro PJ, Clevelario AL, Padilha GA (2017). Bosutinib therapy ameliorates lung inflammation and fibrosis in experimental silicosis. Front Physiol.

[34] Miao R, Ding B, Zhang Y (2016). Proteomic profiling change during the early development of silicosis disease. J Thorac Dis.

[35] Castranova V, Vallyathan V (2000). Silicosis and coal workers' pneumoconiosis. Environ Health Perspect.

[36] Leung CC, Yu IT, Chen W (2012). Silicosis. Lancet.

[37] Booton R, Lindsay MA (2014). Emerging role of MicroRNAs and long noncoding RNAs in respiratory disease. Chest.

[38] Zhao Y, Hao C, Bao L (2020). Silica particles disorganize the polarization of pulmonary macrophages in mice. Ecotoxicol Environ Saf.

[39] Yokohira M, Hashimoto N, Yamakawa K (2009). Lack of modifying effects of Intratracheal instillation of quartz or dextran sulfate sodium (DSS) in drinking water on lung tumor development initiated with 4-(Methylnitrosamino)-1-(3-pyridyl)-1-butanone (NNK) in female a/J mice. J Toxicol Pathol.

[40] Li LJ, Zhao W, Tao SS (2017). Comprehensive long non-coding RNA expression profiling reveals their potential roles in systemic lupus erythematosus. Cell Immunol.

[41] Liu Y, Li Y, Xu Q (2018). Long non-coding RNA-ATB promotes EMT during silica-induced pulmonary fibrosis by competitively binding miR-200c. Biochim Biophys Acta Mol basis Dis.

[42] Freeley S, Kemper C, Le Friec G (2016). The "ins and outs" of complement-driven immune responses. Immunol Rev.

[43] Gialeli C, Gungor B, Blom AM (2018). Novel potential inhibitors of complement system and their roles in complement regulation and beyond. Mol Immunol.

[44] Sorokin L (2010). The impact of the extracellular matrix on inflammation. Nat Rev Immunol.

[45] Faxuan W, Qin Z, Dinglun Z (2012). Altered microRNAs expression profiling in experimental silicosis rats. J Toxicol Sci.

[46] Girolami A, Cosi E, Ferrari S (2018). Heparin, coumarin, protein C, antithrombin, fibrinolysis and other clotting related resistances: old and new concepts in blood coagulation. J Thromb Thrombolysis.

[47] Chistiakov DA, Orekhov AN, Bobryshev YV (2015). Vascular smooth muscle cell in atherosclerosis. Acta Physiol (Oxford).

[48] Clarke MC, Talib S, Figg NL (2010). Vascular smooth muscle cell apoptosis induces interleukin-1-directed inflammation: effects of hyperlipidemia-mediated inhibition of phagocytosis. Circ Res.

[49] Ohta H, Chiba S, Ebina M (2012). Altered expression of tight junction molecules in alveolar septa in lung injury and fibrosis. Am J Phys Lung Cell Mol Phys.

[50] Wong TL, Che N, Ma S (2017). Reprogramming of central carbon metabolism in cancer stem cells. Biochim Biophys Acta Mol basis Dis.

[51] IARC Working Group on the Evaluation of Carcinogenic Risks to Humans. Arsenic, metals, fibres, and dusts. IARC Monogr Eval Carcinog Risks Hum. 2012;100(Pt C):11–465. PMC4781271 23189751

[52] Sato T, Shimosato T, Klinman DM (2018). Silicosis and lung cancer: current perspectives. Lung Cancer (Auckl).

[53] Bayram S, Fürst S, Forbes M (2020). Analysing central metabolism in ultra-high resolution: at the crossroads of carbon and nitrogen. Mol Metab.

