# Supplementary material for: Dynamic assessing silica particle-induced pulmonary fibrosis and associated regulation of long non-coding RNA expression in Wistar rats
Source: Genes Environ. 2021 Jun 15;43:23. doi: 10.1186/s41021-021-00193-3 (PMC8204564; doi:10.1186/s41021-021-00193-3)
Supplement: Supplementary file 1 — Additional file 1: Fig. S1. Heat map showing significant differentially expressed lncRNAs in lungs of rat model of pulmonary fibrosis. a: [1S3], [1S1] and [1S2] represent pulmonary fibrosis samples on the first day; [1C1], [1C2] and [1C3] represent control samples. b: Silica-exposed group and control samples on 7th day. c: Silica-exposed group and control samples on 14th day. d: Silica-exposed group and control samples on 21th day. e: Silica-exposed group and control samples on 28th day. Each row represents a lncRNA and each column represents a sample. Dendrograms produced by clustering analysis of the samples are shown on the top. The red represent up-regulated lncRNAs and blue represent down-regulated lncRNAs in lungs of rat model of pulmonary fibrosis compared to control. Fig. S2. The data from lung tissues of rats of silica-induced was sampled at five time points (day 1, 7, 14, 21 and 28). The colored profiles had a statistically significant number of genes assigned. Non-white profiles of the same color represent profiles grouped into a single cluster. Table S1. The top 10 up-regulated and down-regulated lncRNAs in lungs of silica-induced rats on the first day. Table S2.. The top 10 up-regulated and down-regulated lncRNAs in lungs of silica-induced rats on the 7th day. Table S3. The top 10 up-regulated and down-regulated lncRNAs in lungs of silica-induced rats on the 14th day. Table S4. The top 10 up-regulated and down-regulated lncRNAs in lungs of silica-induced rats on the 21th day. Table S5. The top 10 up-regulated and down-regulated lncRNAs in lungs of silica-induced rats on the 28th day. Table S6. The up-regulated and down-regulated mRNAs related to lncRNAs in profile 39 and 8 in lungs of silica-induced rats. [file 41021_2021_193_MOESM1_ESM.doc]

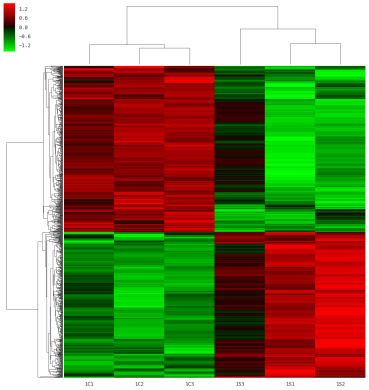

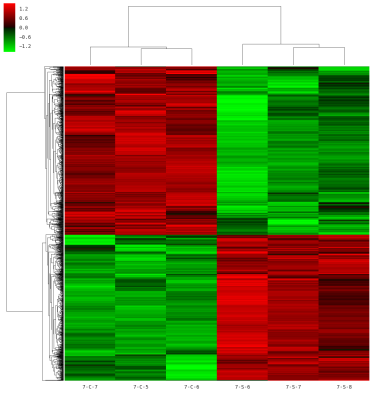

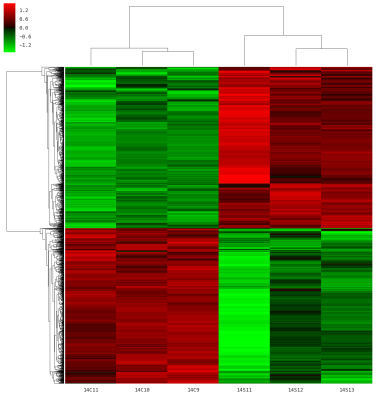


a b c


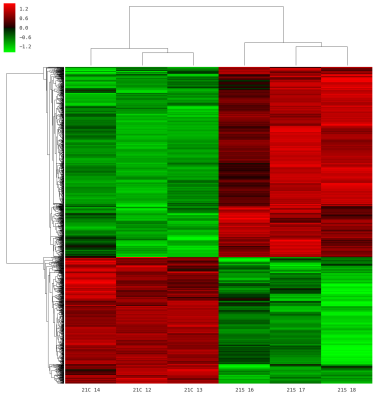

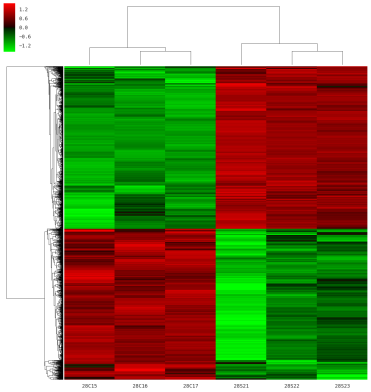


d e

Fig. S1 Heat map showing significant differentially expressed lncRNAs in lungs of rat model of pulmonary fibrosis. a: [1S3], [1S1] and [1S2] represent pulmonary fibrosis samples on the first day; [1C1], [1C2] and [1C3] represent control samples. b: Silica-exposed group and control samples on 7th day. c: Silica-exposed group and control samples on 14th day. d: Silica-exposed group and control samples on 21th day. e: Silica-exposed group and control samples on 28th day. Each row represents a lncRNA and each column represents a sample. Dendrograms produced by clustering analysis of the samples are shown on the top. The red represent up-regulated lncRNAs and blue represent down-regulated lncRNAs in lungs of rat model of pulmonary fibrosis compared to control.





Fig. S2 The data from lung tissues of rats of silica-induced was sampled at five time points (day 1, 7, 14, 21 and 28). The colored profiles had a statistically significant number of genes assigned. Non-white profiles of the same color represent profiles grouped into a single cluster.

Table S1. The top 10 up-regulated and down-regulated lncRNAs in lungs of silica-induced rats on the first day

| Up-regulated lncRNAs | | Down-regulated lncRNAs | |
| --- | --- | --- | --- |
| lncRNAs | Fold change | lncRNAs | Fold change |
| NONRATT008954.2 | 4.4364905 | NONRATT023050.2 | 7.649372 |
| ENSRNOT00000077346.1 | 4.338576 | NONRATT025404.2 | 5.158114 |
| NONRATT016045.2 | 4.0593762 | NONRATT026908.2 | 5.1249223 |
| NONRATT016130.2 | 3.8505647 | NONRATT029477.2 | 4.946014 |
| NONRATT005815.2 | 3.3709428 | NONRATT008985.2 | 4.7944026 |
| NONRATT027205.2 | 3.3156953 | ENSRNOT00000025536 | 4.617265 |
| NONRATT005359.2 | 3.228299 | NONRATT030561.2 | 4.4172773 |
| NONRATT003846.2 | 3.2036881 | NONRATT016087.2 | 4.381305 |
| NONRATT000826.2 | 3.1712434 | NONRATT021716.2 | 4.2227516 |
| NONRATT008570.2 | 3.1699114 | ENSRNOT00000090134.1 | 4.207009 |

Table S2. The top 10 up-regulated and down-regulated lncRNAs in lungs of silica-induced rats on the 7th day

| Up-regulated lncRNAs | | Down-regulated lncRNAs | |
| --- | --- | --- | --- |
| lncRNAs | Fold change | lncRNAs | Fold change |
| NONRATT016029.2 | 23.203049 | NONRATT000767.2 | 8.542871 |
| NONRATT029249.2 | 11.943137 | NONRATT027668.2 | 7.602506 |
| NONRATT030809.2 | 11.707416 | NONRATT017382.2 | 6.6364384 |
| NONRATT010333.2 | 10.541904 | NONRATT007828.2 | 6.3603334 |
| NONRATT021486.2 | 9.490502 | NONRATT029824.2 | 5.7557225 |
| NONRATT003404.2 | 9.139105 | NONRATT029231.2 | 5.4184895 |
| NONRATT016030.2 | 8.72531 | NONRATT028521.2 | 4.853228 |
| NONRATT020589.2 | 8.202733 | NONRATT028682.2 | 4.5890284 |
| ENSRNOT00000084386.1 | 7.791705 | NONRATT004740.2 | 4.580962 |
| ENSRNOT00000088727.1 | 7.6722174 | NONRATT027842.2 | 4.4284225 |

Table S3. The top 10 up-regulated and down-regulated lncRNAs in lungs of silica-induced rats on the 14th day

| Up-regulated lncRNAs | | Down-regulated lncRNAs | |
| --- | --- | --- | --- |
| lncRNAs | Fold change | lncRNAs | Fold change |
| NONRATT030809.2 | 11.2041025 | NONRATT000635.2 | 7.646588 |
| NONRATT009935.2 | 10.097702 | NONRATT027668.2 | 7.0156207 |
| NONRATT020589.2 | 9.975832 | ENSRNOT00000037639 | 5.5938754 |
| ENSRNOT00000080005.1 | 9.631212 | NONRATT004687.2 | 5.502133 |
| ENSRNOT00000033123 | 7.9503903 | NONRATT029824.2 | 5.379213 |
| NONRATT012026.2 | 7.1873 | NONRATT002728.2 | 5.27119 |
| NONRATT029249.2 | 7.1789017 | NONRATT000767.2 | 4.7600217 |
| NONRATT025398.2 | 6.964083 | ENSRNOT00000073481 | 4.423144 |
| ENSRNOT00000056608 | 6.617643 | NONRATT002790.2 | 4.339205 |
| NONRATT020346.2 | 6.541318 | NONRATT028142.2 | 4.321625 |

Table S4. The top 10 up-regulated and down-regulated lncRNAs in lungs of silica-induced rats on the 21th day

| Up-regulated lncRNAs | | Down-regulated lncRNAs | |
| --- | --- | --- | --- |
| lncRNAs | Fold change | lncRNAs | Fold change |
| NONRATT030809.2 | 10.446728 | NONRATT022370.2 | 6.635508 |
| NONRATT029249.2 | 9.914126 | NONRATT016541.2 | 6.503894 |
| NONRATT020346.2 | 9.446369 | NONRATT026369.2 | 6.2682242 |
| NONRATT012026.2 | 8.312622 | NONRATT001596.2 | 5.94666 |
| ENSRNOT00000084080.1 | 7.7981834 | NONRATT020262.2 | 5.0459757 |
| NONRATT021934.2 | 7.70821 | ENSRNOT00000092197.1 | 4.962062 |
| NONRATT018155.2 | 7.5613008 | NONRATT028785.2 | 4.7664013 |
| ENSRNOT00000033123 | 7.4815736 | ENSRNOT00000007070 | 4.6680465 |
| NONRATT010849.2 | 7.4562087 | NONRATT020698.2 | 4.6549864 |
| NONRATT005042.2 | 7.245416 | NONRATT005334.2 | 4.4012513 |

Table S5. The top 10 up-regulated and down-regulated lncRNAs in lungs of silica-induced rats on the 28th day

| Up-regulated lncRNAs | | Down-regulated lncRNAs | |
| --- | --- | --- | --- |
| lncRNAs | Fold change | lncRNAs | Fold change |
| NONRATT025712.2 | 18.001942 | NONRATT029824.2 | 8.038739 |
| ENSRNOT00000078895.1 | 12.259474 | NONRATT029477.2 | 6.5919914 |
| NONRATT020346.2 | 11.373866 | NONRATT005923.2 | 6.582022 |
| ENSRNOT00000033123 | 10.99439 | NONRATT004687.2 | 6.1523004 |
| NONRATT021934.2 | 10.601546 | NONRATT000710.2 | 5.776317 |
| NONRATT021486.2 | 10.540024 | NONRATT000767.2 | 5.7112455 |
| NONRATT030809.2 | 10.484751 | ENSRNOT00000091438.1 | 5.669223 |
| ENSRNOT00000071577 | 10.384734 | NONRATT008985.2 | 5.5762234 |
| NONRATT029249.2 | 10.098548 | NONRATT029231.2 | 5.5511885 |
| NONRATT012026.2 | 9.739349 | NONRATT005156.2 | 5.4823456 |

Table S6: The up-regulated and down-regulated mRNAs related to lncRNAs in profile 39 and 8 in lungs of silica-induced rats

| Genes name | profile | regulation |
| --- | --- | --- |
| Akr1b8 | 39 | up |
| Bard1 | 39 | up |
| Ctsd | 39 | up |
| Dhrs9 | 39 | up |
| Fermt3 | 39 | up |
| Ftl1 | 39 | up |
| Galns | 39 | up |
| Itgam | 39 | up |
| Kng1 | 39 | up |
| Msr1 | 39 | up |
| Naip5 | 39 | up |
| P2rx4 | 39 | up |
| Parvg | 39 | up |
| Pkm | 39 | up |
| Sgms2 | 39 | up |
| Slc38a1 | 39 | up |
| Slc4a5 | 39 | up |
| Slc7a7 | 39 | up |
| Tfeb | 39 | up |
| Tiam1 | 39 | up |
| Trem2 | 39 | up |
| Uap1l1 | 39 | up |
| Vcan | 39 | up |
| Vegfb | 39 | up |
| Xylt1 | 39 | up |
| Acer2 | 8 | down |
| Aldh1a1 | 8 | down |
| Amotl1 | 8 | down |
| Amotl2 | 8 | down |
| Arap3 | 8 | down |
| Calcrl | 8 | down |
| Car4 | 8 | down |
| Glp1r | 8 | down |
| RT1-T24-1 | 8 | down |
| Sema3b | 8 | down |
| Slc29a1 | 8 | down |
| Tead4 | 8 | down |
| Tfpi | 8 | down |
| Thra | 8 | down |
| Tnfsf10 | 8 | down |
